# Supplementary material for: Comprehensive Annotation and Functional Exploration of MicroRNAs in Lettuce
Source: Front Plant Sci. 2021 Dec 24;12:781836. doi: 10.3389/fpls.2021.781836 (PMC8739914; doi:10.3389/fpls.2021.781836)
Supplement: Supplementary file 2 [file Data_Sheet_8.ZIP › GSM2230747.plot/Lsa-miR477a_Lsat_1_v5_gn_3_90761.1_2129_TPlot.pdf]

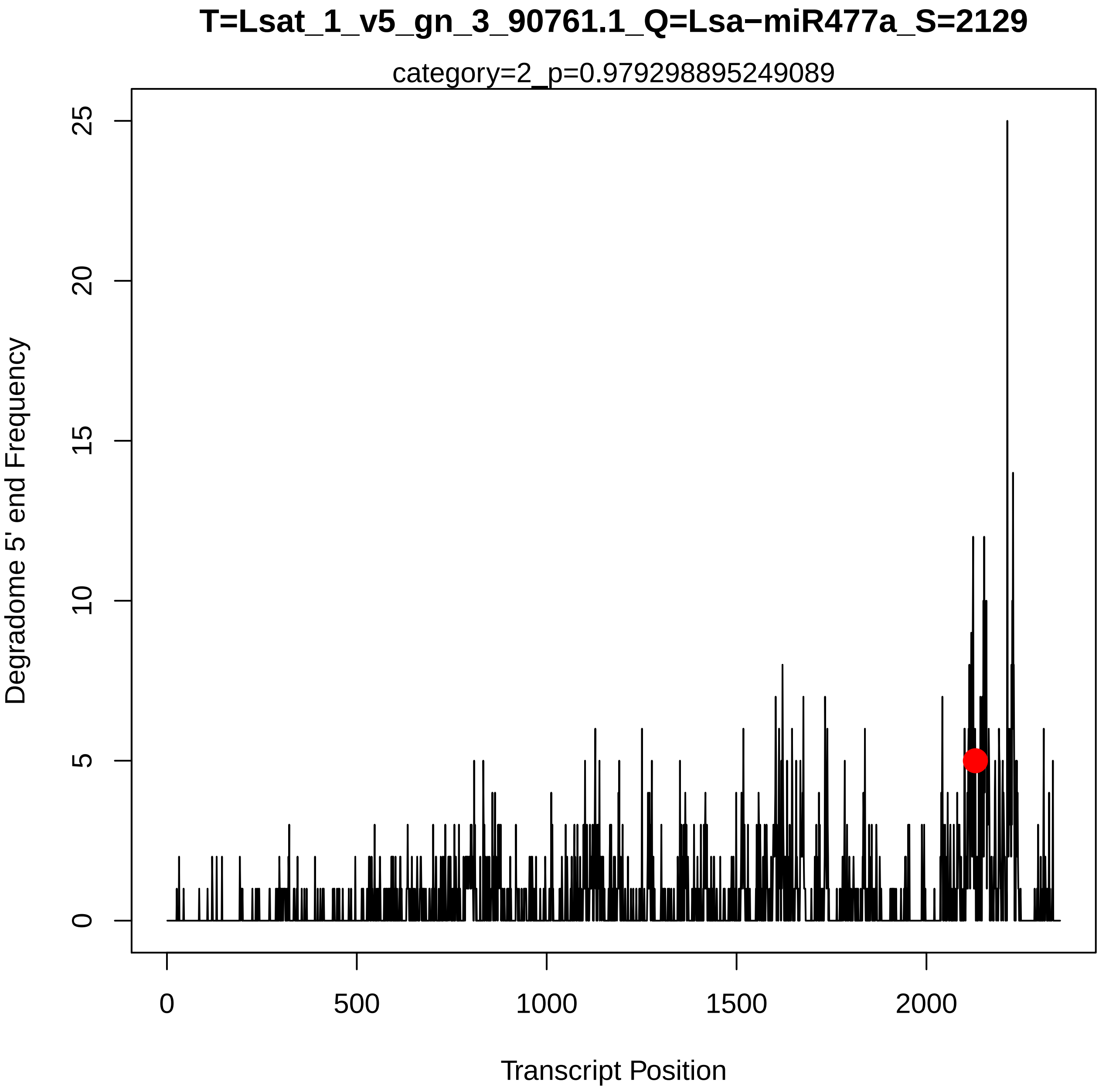

T=Lsat_1_v5_gn_3_90761.1_Q=Lsa−miR477a_S=2129
category=2_p=0.979298895249089
Degradome 5' end Frequency
Transcript Position
0
5
10
15
20
25
0
500
1000
1500
2000
